# *Platycodon grandiflorum* Root Protects against Aβ-Induced Cognitive Dysfunction and Pathology in Female Models of Alzheimer’s Disease

**DOI:** 10.3390/antiox10020207

**Published:** 2021-02-01

**Authors:** Yunkwon Nam, Soo Jung Shin, Yong Ho Park, Min-Jeong Kim, Seong Gak Jeon, Hyewon Lee, Yeji Choi, Tae-Jin Kim, Seong Min Shin, Jwa-Jin Kim, Doo-Han Yoo, Hyung Don Kim, Sujin Kim, Minho Moon

**Affiliations:** 1 Department of Biochemistry, College of Medicine, Konyang University, 158, Gwanjeodong-ro, Seo-gu, Daejeon 35365, Korea; yunkwonnam@gmail.com (Y.N.); tlstnzz@konyang.ac.kr (S.J.S.); znf900809@naver.com (Y.H.P.); rlaalswjd403@naver.com (M.-J.K.); jsg7394@naver.com (S.G.J.); ssminhk@gmail.com (S.M.S.); 2 Department of Neural Development and Disease, Korea Brain Research Institute (KBRI), Daegu 41068, Korea; 3 Department of Occupational Therapy, Konyang University, 158, Gwanjeodong-ro, Seo-gu, Daejeon 35365, Korea; lavine-woni@hanmail.net (H.L.); yeji.choi12@gmail.com (Y.C.); kaiser0022@naver.com (T.-J.K.); glovia@konyang.ac.kr (D.-H.Y.); 4 Departments of Nephrology, School of Medicine, Chungnam National University, Daejeon 35015, Korea; kjj1021@naver.com; 5 Research Institute for Dementia Science, Konyang University, Daejeon 35365, Korea; 6 Department of Herbal Crop Research, NIHHS, Eumsung 27709, Korea

**Keywords:** Alzheimer’s disease, amyloid beta, *Platycodon grandiflorum*, cognitive dysfunction, 5XFAD mice

## Abstract

Alzheimer’s disease (AD) is a devastating neurodegenerative disease characterized by irreversible cognitive dysfunction. Amyloid beta (Aβ) peptide is an important pathological factor that triggers the progression of AD through accumulation and aggregation, which leads to AD-related pathologies that consequently affect cognitive functions. Interestingly, several studies have reported that *Platycodon grandiflorum* root extract (PGE), besides exhibiting other bioactive effects, displays neuroprotective, anti-neuroinflammatory, and cognitive-enhancing effects. However, to date, it is not clear whether PGE can affect AD-related cognitive dysfunction and pathogenesis. Therefore, to investigate whether PGE influences cognitive impairment in an animal model of AD, we conducted a Y-maze test using a 5XFAD mouse model. Oral administration of PGE for 3 weeks at a daily dose of 100 mg/kg significantly ameliorated cognitive impairment in 5XFAD mice. Moreover, to elucidate the neurohistological mechanisms underlying the PGE-mediated alleviative effect on cognitive dysfunction, we performed histological analysis of hippocampal formation in these mice. Histopathological analysis showed that PGE significantly alleviated AD-related pathologies such as Aβ accumulation, neurodegeneration, oxidative stress, and neuroinflammation. In addition, we observed a neuroprotective and antioxidant effect of PGE in mouse hippocampal neurons. Our findings suggest that administration of PGE might act as one of the therapeutic agents for AD by decreasing Aβ related pathology and ameliorating Aβ induced cognitive impairment.

## 1. Introduction

Alzheimer’s disease (AD) is the most prevalent type of dementia, and its prevalence is increasing worldwide [1]. The major neuropathologic criteria of AD are the formation of extracellular amyloid-beta (Aβ) plaques precipitated by Aβ aggregation and intracellular neurofibrillary tangles produced by abnormal hyperphosphorylation of tau proteins [2]. In particular, dysregulation of Aβ levels causes several AD-related pathologies, such as the formation of Aβ plaques, neurodegeneration, neuroinflammation, mitochondrial dysfunction, and impairment of adult neurogenesis [1,3]. In addition, aggregation of Aβ results in the activation of apoptosis signal-regulating kinase 1 (ASK1) to cause neuronal cell death and overstimulation of N-methyl-D-aspartate (NMDA) receptors to induce synaptic degeneration [4,5]. Moreover, Aβ-induced activated microglia release neurotoxic factors, such as reactive oxygen species (ROS), pro-inflammatory cytokines, and chemokines, to provoke neuronal damage. Subsequently, neuronal damage caused by pro-inflammatory cytokines may secrete microglia activators such as MMP3 and laminin, thus forming a self-perpetuating cycle that reactivates the microglia [6,7]. In addition, the localization of Aβ in mitochondrial membranes destroys the electron transport chain and increases the production of ROS, causing mitochondrial and neuronal dysfunction [8]. Furthermore, an imbalance of neurotransmitter release between glutamatergic and GABAergic system-induced Aβ contributes to impaired adult hippocampal neurogenesis (AHN) [9]. These alterations implied that Aβ directly or indirectly leads to neuronal cell death and synaptic degeneration, which is closely correlated with a cognitive impairment and memory loss [10,11]. Thus, various Aβ-related pathologies ultimately provoke cognitive decline in individuals with AD.

*Platycodon grandiflorum* (PG), one of the well-known traditional herbal remedies found in East Asia, including China, Korea, and Japan, has been widely used because it not only exhibits valuable bioactive effects but also does not have toxicity and adverse effects [12]. A number of studies have reported that PG has a variety of bioactive functions, including antioxidant, anti-inflammatory, anti-obesity, anti-diabetic, and anti-tumor activities [13,14,15,16,17]. In addition, PG shows considerable beneficial effects, such as anti-cholesterol effects, reduced levels of low-density lipoproteins, and increased coronary blood flow in the metabolic and cardiovascular systems [12]. In diseases with various pathological features, such as AD, natural products, especially PG, which are multi-targeting and multi-functional, may have the potential to be used as therapeutic agents. Notably, *Platycodon grandiflorum* root extract (PGE) and its components have been reported to exhibit neuroprotective, anti-neuroinflammatory, and cognitive-enhancing effects in various disease models [18,19,20,21,22,23,24,25]. In particular, PGE has shown neuroprotective effects against Aβ-induced cytotoxicity [18]. In addition, PGE is known to inhibit L-glutamate-induced neuronal cell death, H_2_O_2_-induced membrane damage, and intracellular ROS formation in vitro [19,20,21]. Moreover, PGE and its components, such as platycodin D and platycoside E, exhibit anti-inflammatory effects through the inhibition of LPS-induced COX-2 and iNOS expression, NF-κB activation, and ROS and pro-inflammatory cytokine production from microglial cells [22,23]. Furthermore, PGE ameliorates ethanol-induced memory impairment and scopolamine-induced amnesia in mice [24,25]. Therefore, it can be speculated that PGE is a potential therapeutic agent that can be used to treat a variety of pathophysiologies of AD. However, there have been no studies examining the therapeutic effects of PGE on Aβ-induced cognitive dysfunction and pathology in animal models of AD.

In this study, we hypothesized that PGE with neuroprotective, antioxidant, anti-neuroinflammatory, and cognitive-enhancing effects can be effective in AD treatment with various pathological features. However, there is currently little to no research on the efficacy of PGE on AD pathogenesis, and it is unclear whether PGE can affect AD-related cognitive impairments and pathologies. Thus, we investigated the therapeutic effects of PGE on Aβ-induced cognitive dysfunction and AD-related neurohistopathological changes, such as Aβ deposition, neurodegeneration, oxidative stress, neuroinflammation, and impairment of adult hippocampal neurogenesis in female 5XFAD mice. These are transgenic mice over-expressing mutant human amyloid precursor protein (APP695) with the Swedish (K670N, M671L), Florida (I716V), and London (V717I) mutations along with human presenilin 1 (PSEN1) harboring two (M146L and L286V) mutations, all known to accelerated accumulation of Aβ and development of early onset familial AD (FAD). In the 5XFAD mice, AD-related phenotypes aggressively appear from an early age, and amyloid plaques with gliosis are detected at 2 months of age [26]. In addition, the Aβ pathology in 5XFAD mice is more severe in females than in males [27], and cognitive decline in 5XFAD mice begins at ages of 6 months [28,29]. In addition, we examined the antioxidant effect of PGE in Aβ-treated mouse hippocampal neuron cell line (HT22).

## 2. Materials and Methods

### 2.1. Sample Preparation

The roots of a 3-year-old PG cv. Eutteumbaek plant were harvested from Boeun, Chungcheongbuk-do, Korea, in 2018. They were then air-dried at 55 °C for 72 h. To prepare the total PGE, 50 L of water was added per 5 kg of ground roots and extracted at 90 °C for 6 h using an extraction machine (COSMOS-660, Kyungseo E&P Co., Ltd., Incheon, Korea). The extract was filtered, and the supernatant was lyophilized to prepare the extracted dry basis. The extraction yield was 34%. The sample was stored at −80 °C until analysis.

### 2.2. Analysis of Platycoside E and Platycodin D Using HPLC-ELSD

Standards of platycoside E and platycodin D were purchased from Cheongdu Biopurify Phytochemical Ltd. (Cheongdu, Sichuan, China). In order to perform quantitative analysis of platycoside E and platycodin D, 0.5 g of the extracted dry basis of *Platycodon grandiflorum* roots was dissolved in 40 mL of distilled water and defatted with diethyl ether in a separatory funnel. The aqueous layer was extracted with 40 mL of water-saturated n-butanol, three times. The n-butanol layer was evaporated at 50 °C, and the resulting residue was dissolved in methanol, and subjected to analysis. Platycoside E and platycodin D were identified by matching the retention time against the standards, and their contents were determined using calibration curves (Table S1). Platycoside E and platycodin D contents from PGE were analyzed using high-performance liquid chromatography (HPLC) with an evaporative light scattering detector (ELSD) (Waters Alliance 2695 HPLC system with 2424 ELSD, Waters, Milford, MA, USA), and a C-18 column (Luna C-18, Phenomenox, 250 × 4.6 mm, 5 μm, Torrance, CA, USA) was used for chromatography. A gradient of mobile phase was generated using (A) water and (B) acetonitrile as follows: 0–3 min, 21–21% B; 3–23 min, 21–23% B; 23–38 min, 23–24% B; 38–70 min, 24–100% B; 70–75 min, 100–100% B. The flow rate was 1.0 mL/min, the sample injection volume was 30 μL, and the column temperature was 40 °C. The ELSD conditions were as follows: Nebulizer temperature, 42 °C; drift tube temperature, 85 °C; and N_2_ gas pressure, 50 psi.

### 2.3. Animals and PGE Administration

The 5XFAD mouse (Tg6799; Jackson Laboratory, Bar Harbor, ME, USA) expresses five mutations related to the early onset of familial AD. The five mutations comprise human PSEN1 mutation with M146L and L286V, and human APP mutation with Swedish (K607N and M671L), Florida (I716V), and London (V717I). Wild-type (WT) mice were obtained by crossing female B6SJL/F1 and male 5XFAD mice. Mice were classified as WT and 5XFAD through genotyping of tail DNA (APP forward: 5-AGG ACT GAC CAC TCG ACC AG-3, APP reverse: 5-CGG GGG TCT AGT TCT GCA T-3, PSEN1 forward: 5-AAT AGA GAA CGG CAG GAG CA-3, PSEN1 reverse: 5-GCC ATG AGG GCA CTA ATC AT-3) (Table S2). The PGE was dissolved in saline before oral injection and administered to 6-month-old female WT and 5XFAD mice for 3 weeks at a dose of 100 mg/kg daily [30]. The behavioral experiments were repeated twice to improve statistical precision. We used a total of 12–13 animals to demonstrate the effect of PGE on cognitive decline. Animals were randomly divided into four groups for first behavioral trials: (1) WT + vehicle group (*n* = 4) treated with saline, (2) WT + PGE group (*n* = 5) treated with PGE, (3) 5XFAD + vehicle group (*n* = 5) treated with saline, and (4) 5XFAD + PGE group (*n* = 5) treated with PGE. Animals were randomly divided into four groups for repetition of behavioral test: (1) WT + vehicle group (*n* = 8) treated with saline, (2) WT + PGE group (*n* = 8) treated with PGE, (3) 5XFAD + vehicle group (*n* = 8) treated with saline, and (4) 5XFAD + PGE group (*n* = 8) treated with PGE. The first trial groups were used for histopathological analysis. Animal experiments were conducted according to the Guide for the Care and Use of Laboratory Animals (National Institutes of Health publication No. 85–23, revised 1985) for maintenance, care, and treatment and was approved by the Institutional Animal Care and Use Committee of Konyang University (Project identification code: P-20-15-E-01, date: 27 April 2020).

### 2.4. Behavioral Test

A short-term spatial recognition memory test using the Y-maze was conducted as described in a previous study [31]. In the experimental environment, the temperature and humidity were maintained at 23 ± 1 °C and 60 ± 10%, respectively. The three arms of the Y-maze (each 8 cm wide× 30 cm long × 15 cm tall) are located at an angle of 120° [30]. To measure the memory function, mice were first allowed to adapt themselves to the experimental environment for a week before the behavioral experiment. For a week before the behavioral experiment, every day the mice were individually placed for an hour in a dark environment with identical environmental factors to that of the experiment, and then handled for 10 min before being placed for 5 min in the Y-maze where they could get accustomed to the unfamiliar environment. The mice are placed in the Y-maze and habituated until anxiety and freezing response disappear. After the last PGE administration, a behavior test using the Y-maze was conducted. Each mouse was placed in the center of the Y-maze and allowed to explore it freely. The total arm entries (locomotor activity) and spontaneous alternation were manually measured for each mouse for 8 min by two experimenters. Spontaneous alternations were defined as sequential entries into the three arms (i.e., ABC, BAC, or CBA) without repeated entries (i.e., ABA, or CBC). The percentage of alternations was calculated as follows: [(the number of alterations)/(the total number of arms entered−2)] × 100.

### 2.5. Preparation of Brain Tissue

After the behavioral experiment, the mice were anesthetized and treated by sequential cardiac-perfusion with 0.05 M phosphate-buffered saline (PBS) and 4% paraformaldehyde (PFA) solution prepared by dissolving PFA in 0.1 M PBS. The brains were removed, post-fixed in 4% PFA solution for 20 h at 4 °C and immersed in 30% sucrose solution prepared in 0.05 M PBS for cryoprotection. Next, each brain was cut into coronal sections at a thickness of 30 μm using a CM1850 cryostat at −25 °C (Leica Biosystems, Wetzlar, Germany). The cut tissues were stored in a storing solution (cryoprotectant) containing 25% ethylene glycol and glycerol in 0.05 M phosphate buffer at 4 °C until analyzed immunohistochemically.

### 2.6. Immunofluorescence Labeling

To examine immunoreactivity, three to four brain tissues were cut at intervals of 150–180 μm (−1.46 and −2.06 mm to the bregma) and 270–350 μm (−2.70 and −3.80 mm to the bregma) (Figures S1 and S2). Sections thus obtained were briefly rinsed in PBS containing 0.5 mg/mL BSA and 0.3% Triton X-100 and then incubated overnight at 4 °C with the following primary antibodies: Mouse anti-4G8 antibody (1:2000; BioLegend, San Diego, CA, USA), mouse anti-neuronal nuclei (NeuN) antibody (1:500; Merck KGaA, Darmstadt, Germany), mouse anti-synaptophysin (SYN) antibody (1:500; Sigma-Aldrich, St. Louis, MO, USA), mouse anti-4 hydroxynonenal antibody (4 HNE) antibody (1:200; Abcam, Cambridge, MA, USA), goat anti-ionized calcium binding adapter molecule 1 (Iba1) antibody (1:1000; Abcam, Cambridge, MA, USA), and rat anti-glia fibrillary acidic protein (GFAP) antibody (1:500; Thermo Fisher Scientific, Waltham, MA, USA). The brain tissues have treated with 70% formic acid for 20 min for antigen detection before they were incubated with mouse anti-4G8 antibody. Subsequently, the sections were incubated with secondary antibodies for 50–60 min at room temperature (20 to 25 °C). Donkey Alexa 488-conjugated anti-mouse IgG, Donkey Alexa Fluor Plus 488-conjugated anti-mouse IgG, and donkey Alexa 594-conjugated anti-mouse IgG (1:200; Thermo Fisher Scientific, Waltham, MA, USA) were prepared at appropriate dilutions in PBS containing 0.3% Triton X-100. Immunostained sections were mounted on slides using Fluoroshield™ with 4,6-diamidino-2-phenylindole (DAPI) and overlaid with coverslips. 

### 2.7. Immunoperoxidase Labeling

To examine AHN in the brain, sections of −1.70 to −2.18 mm from the bregma were obtained from three to four brain tissues of the mice at 120 to 180 μm intervals. Next, the brain sections were rinsed in PBS and treated with PBS containing 1% H_2_O_2_ for 15 min at room temperature. The sections were then incubated overnight with primary antibodies at 4 °C goat anti-doublecortin (DCX) antibody (1:1000; Santa Cruz Biotechnology, Dallas, TX, USA) and rabbit anti-Ki67 antibody (1:2000; Abcam, Cambridge, MA, USA). Subsequently, the sections were treated with biotinylated secondary antibody (1:200; Vector Laboratories, Burlingame, CA, USA) for one h at room temperature. They were later visualized using an avidin–biotin–peroxidase complex (ABC) solution kit (Vector Laboratories, Burlingame, CA, USA) with urea hydrogen peroxide tablets (Sigma-Aldrich, St. Louis, MO, USA) and DAB (3,3-diaminobenzidine) tablets. After the brain sections were mounted on slides and dehydrated with alcohol and xylene, they were mounted using a mounting medium (Thermo Fisher Scientific, Waltham, MA, USA) and overlaid with cover-slips.

### 2.8. Cell Viability Assay in HT22 Cells

HT22 cells were cultivated in Dulbeccos Modified Eagles Media (DMEM) (WelGENE, Gyeongsan-si, Korea). It was complemented with 10% fetal bovine serum (WelGENE, Gyeongsan-si, Korea) and 100 units/mL penicillin-streptomycin (Gibco, Waltham, MA, USA) and incubated in a humidified 5% CO_2_ atmosphere at 37 °C. The HT22 cells were counted and seeded at 1 × 10^4^ cells/well onto a 96 well microplate. After 24 h, PGE extracts were added at different concentrations (50, 100, 200 ug/mL) for 1 h then treated with Aβ_25–35_ (10 mM; Sigma-Aldrich, St. Louis, MO, USA). After 48 h, the degree of cell viability was quantified using 3-(4,5-dimethylthiazol-2-yl)-5-(3-carboxymethoxyphenyl)-2-(4 sulfophenyl)-2H-tetrazolium (MTS) assay following the manufacturer’s protocol. The absorbance was measured at 490 nm using a multimode microplate reader (Biotek Inc., Winooski, VT, USA). The experiment was carried out in triplicate.

### 2.9. Intracellular ROS Generation in HT22 Cells

Intracellular ROS generation was measured by a modified dichloro-dihydro-fluorescein diacetate (DCFH-DA) assay. The HT22 cells were counted and seeded in black 96-well plates at a density of 1.0 × 10^4^ cells/well and incubated for 24 h. After incubating with extracts for another 24 h, the cultured cells were treated with 10 mM Aβ_25–35_ in serum-free medium (SFM) for 30 min and then with 20 μM DCF-DA in SFM for 30 min; samples were then washed, and 100 μL Dulbecco’s phosphate-buffered saline (DPBS) was added to each well. The fluorescence was measured with a multi-plate reader at 485 nm/535 nm. The experiment was carried out in triplicate.

### 2.10. Image Acquisition and Analysis

To quantify immunoreactivity, images were acquired from the immunostained brain sections using Zeiss LSM 700 (Carl Zeiss AG, Oberkochen, Germany) and Olympus CX23 (Olympus Corporation, Tokyo, Japan) microscopes and analyzed using ImageJ software. The analysis was conducted in the hippocampal formation of the brain sections in each animal (*n* = 4–5). To measure 4G8 immunoreactivity, the area fractions (%), average plaque size (μm^2^), and plaque number per mm^2^ for immune-positive signals in the dorsal subiculum were quantified. Immunoreactivity of NeuN, Iba1, and GFAP was quantified as the number of positive cells per mm^2^ of the dorsal subiculum. In addition, the immunoreactivity of Iba1 and GFAP was quantified as the area fraction of immuno-positive signals in the dorsal subiculum. SYN and 4 HNE immunoreactivity were quantified as the fluorescence intensity (optical density) in the hippocampus. Moreover, immunoreactivity of DCX and Ki67 was quantified as the number of positive cells per length (mm) of the subgranular zone (SGZ) in the hippocampal dentate gyrus (DG), as previously described [32]. Histological quantification, statistical analysis, and image acquisition were performed in a blinded manner for the 4 groups.

### 2.11. Statistical Analysis

All analyses were randomly performed in a blinded manner for the 4 groups. All statistical analyses were performed using GraphPad Prism 7.0 software (GraphPad Software, Inc., La Jolla, CA, USA). All data are shown as the mean ± standard error of the mean (SEM). The significance of differences between the two groups was determined using an independent t-test. Statistical analysis between four groups was performed by one-way ANOVA, followed by Tukey’s post hoc test. Differences were considered statistically significant at *p* < 0.05.

## 3. Results

### 3.1. Standardization of Indicator Components in PGE Using HPLC

**Figure 1:**
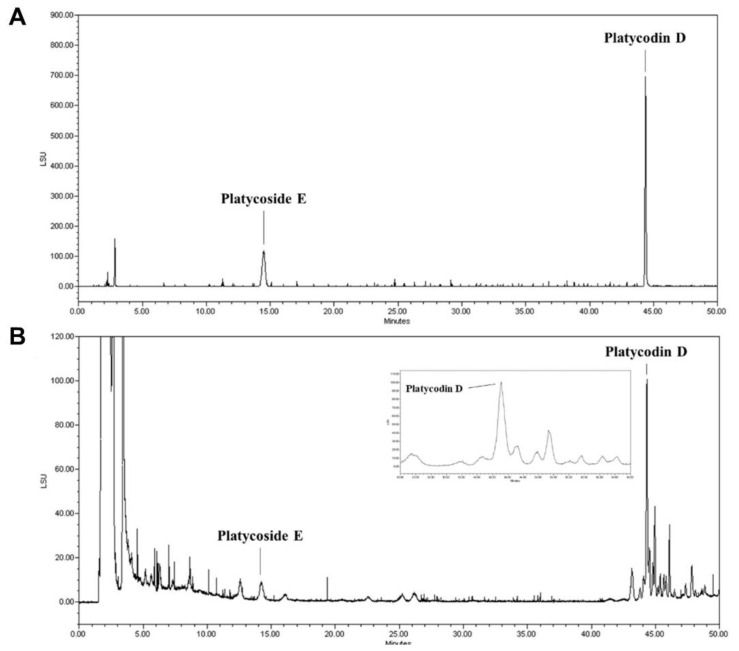
High-performance liquid chromatography with evaporative light scattering detection (HPLC-ELSD) analysis of the *Platycodon grandiflorum* root extract (PGE). (**A**) HPLC chromatograms showed the retention times and peaks of a standard solution containing platydoside E and platycodin D. (**B**) HPLC profile of the PGE extract.

Among the diverse saponins, platycodin D and platycoside E are regarded as the major bioactive molecules and indicator components of PGE [33]. We determined the contents of platycodin D and platycoside E in our sample using HPLC with ELSD; a C-18 column was used for chromatography. The HPLC chromatograms using two standard compounds and our sample are shown (Figure 1A,B). The platycoside E was detected at 14.2 min and platycodin D was detected at 44.3 min. Platycoside E and platycodin D levels were estimated as 1597.14 ± 21.71 and 942.32 ± 97.97 μg/g extract dry basis, respectively, in our sample. Therefore, we confirmed that the indicator components of PGE were present in our sample through chromatographic analysis.

### 3.2. Effect of PGE on Cognitive and Physiological Dysfunction in an Animal Model of AD

**Figure 2:**
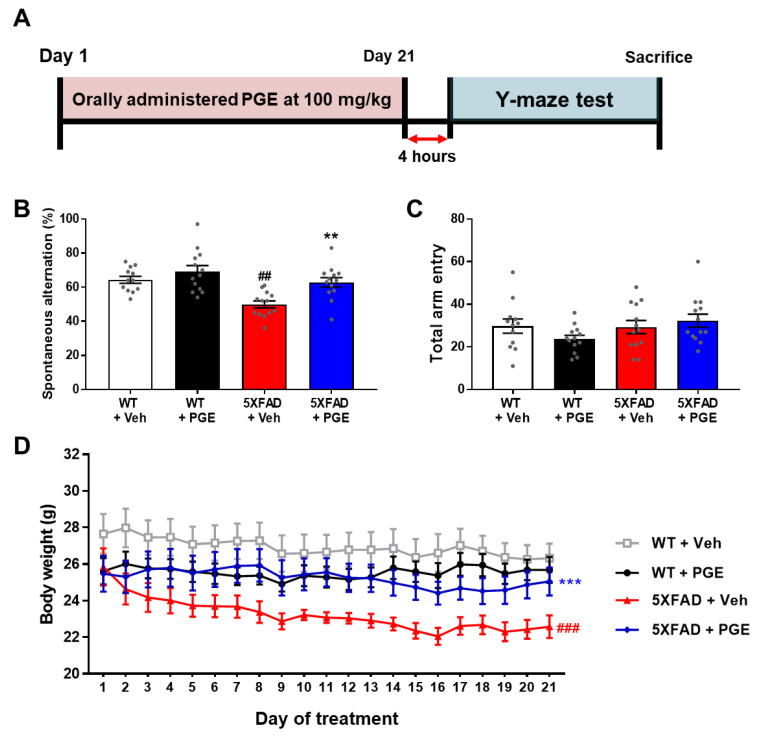
Assessment to determine the attenuating effect of PGE on cognitive and physiological dysfunction in an animal model of Alzheimer’s disease (AD). (**A**) Schematic diagram of the experimental procedure. 100 mg/kg of PGE was orally administered to 5XFAD mice for twenty-one days. Sequentially, after the twenty-first administration, there was a four-hour intermission before the Y-maze test was performed, and then the mice were sacrificed. (**B**) Spontaneous alternations and (**C**) quantitative analysis of total arm entries in wild-type (WT) and 5XFAD group administered with saline and PGE, respectively (*n* = 12–13 mice/group). (**D**) The loss of body weight was alleviated in the PGE-treated 5XFAD mice compared with vehicle-treated 5XFAD mice (*n* = 8 mice/group). The vehicle groups and PGE groups were all treated with the same volume of saline or PGE, respectively. Values are expressed as the mean ± S.E.M. ^##^ *p*< 0.01 and ^###^ *p*< 0.001, vehicle-treated WT mice (white bar) vs. vehicle-treated 5XFAD mice (red bar). ** *p*< 0.01 and *** p< 0.001, vehicle-treated 5XFAD mice (red bar) vs. PGE-treated 5XFAD mice (blue bar). Statistical analysis between four groups was performed by one-way ANOVA, followed by Turkey’s post hoc test.

Although several studies have demonstrated that PGE restores memory loss in ethanol- and scopolamine-induced amnesia models [24,25], there has been no study that PGE has an alleviating effect on Aβ-induced cognitive deficits. Thus, to investigate the effect of PGE on cognitive dysfunction in AD, we performed the Y-maze test in 5XFAD mice, a mouse model of AD administered with PGE. Short-term memory and locomotor activity were evaluated by spontaneous alternation and total arm entry, respectively, in 6-month-old female WT and 5XFAD mice (Figure 2A). Spontaneous alterations in vehicle-treated 5XFAD mice were markedly reduced compared to those in vehicle-treated WT mice. Moreover, PGE-treated 5XFAD mice displayed significantly increased spontaneous alterations compared with vehicle-treated 5XFAD mice (Figure 2B). However, total arm entries were not significantly different between each group (Figure 2C). It is well-known that 5XFAD mice had shown a reduction of body weight in comparison with their WT mice [34]. We measured the body weight of animals during the administration of PGE. The vehicle-treated 5XFAD mice showed a significant decrease in body weight compared to vehicle-treated WT mice. Surprisingly, body weight was significantly increased in PGE-treated 5XFAD mice compared with vehicle-treated 5XFAD mice (Figure 2D). Taken together, these results indicate that oral administration of PGE alleviated the loss of body weight as well as significantly restores Aβ-induced cognitive impairment.

### 3.3. Effect of PGE on Aβ Accumulation in the Aβ-Overexpressing Brain

**Figure 3:**
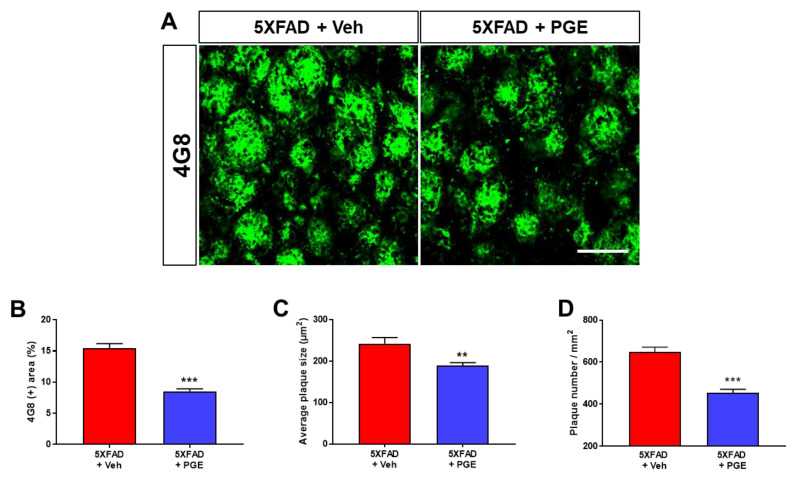
Immunofluorescence staining of the subiculum of 5XFAD AD mouse models to elucidate the inhibitory effect of PGE on Aβ accumulation. (**A**) Representative images show 4G8 immunoreactivity in the subiculum of 5XFAD mice treated with vehicle and PGE. Quantitative analyses of Aβ accumulation are indicated by (**B**) 4G8-positive area, (**C**) average plaque size, and (**D**) plaque number. The vehicle group and PGE group were both treated with the same volume of saline or PGE, respectively. Values are expressed as the mean ± S.E.M. Scale bar = 50 μm. ** *p* < 0.01 and *** *p* < 0.001: Vehicle-treated 5XFAD mice (red bar) vs. PGE-treated 5XFAD mice (blue bar). Statistical analysis between the two groups was performed by an independent t-test (30–32 images of brain section obtained from 5 mice in the 5XFAD + vehicle group and 5 mice in the 5XFAD + PGE group were used for the analysis).

Accumulation of Aβ in brains with AD directly and strongly affects the onset and progression of cognitive decline [35]. Accordingly, to investigate whether PGE administration influenced the accumulation of Aβ, we performed immunofluorescence staining using the 4G8 antibody in the subiculum of 5XFAD mice (Figure 3A). We evaluated Aβ accumulation in vehicle-treated and PGE-treated 5XFAD mice by quantifying the 4G8-positive area, average plaque size, and plaque number. The results demonstrated that 4G8-positive area, average plaque size, and plaque number in 5XFAD mice treated with PGE were significantly reduced compared with those treated with vehicle (Figure 3B–D). Hence, our findings suggest that administration of PGE inhibits Aβ accumulation.

### 3.4. Effect of PGE on Neurodegeneration in the Brain of 5XFAD Mice

**Figure 4:**
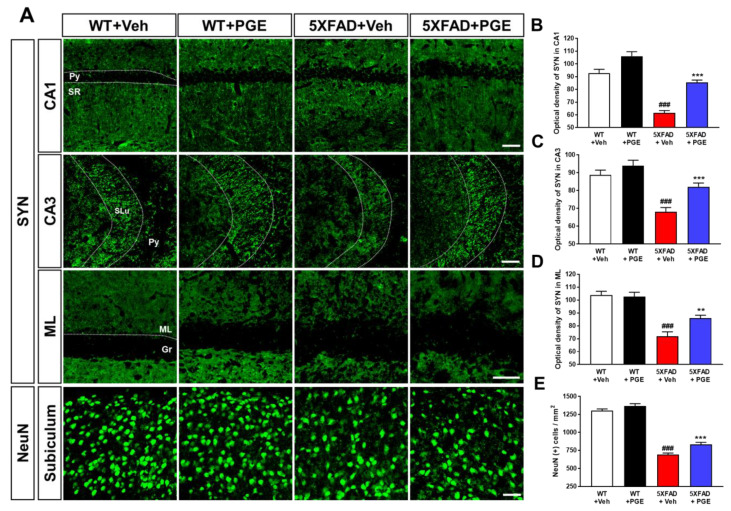
Immunofluorescence staining to demonstrate the protective effect of PGE on neurodegeneration in the hippocampal formation of the 5XFAD mouse model of AD. (**A**) Representative images show neuronal nuclei (NeuN) and synaptophysin (SYN) immunoreactivity in the hippocampal formation in WT and 5XFAD mice treated with vehicle and PGE, respectively. Quantitative analyses of synaptic loss are displayed by the optical density of SYN in (**B**) CA1, (**C**) CA3, and (**D**) ML of the hippocampus. (**E**) Quantitative analysis of neuronal cell death is displayed by NeuN-positive cells in the subiculum. The vehicle groups and PGE groups were all treated with the same volume of dissolved in saline or PGE, respectively. Data are presented as the mean ± S.E.M. Scale bar = 50 μm. ^###^ *p*< 0.001: Vehicle-treated WT mice (white bar) vs. vehicle-treated 5XFAD mice (red bar). ** *p*< 0.01 and *** *p*< 0.001: Vehicle-treated 5XFAD mice (red bar) vs. PGE-treated 5XFAD mice (blue bar). Statistical analysis between the four groups was performed by one-way ANOVA, followed by Turkey’s post hoc test (*n* = 30–32 images of brain section obtained from 4 mice in the WT + vehicle group, 5 mice in the WT + PGE group, 5 mice in the 5XFAD + vehicle group, and 5 mice in the 5XFAD + PGE group were used for the analysis). DG, dentate gyrus; Gr, granular layer; ML, molecular layer; Py, pyramidal tract; SLu, stratum lucidum; SR, stratum radiatum.

Neurodegeneration, including neuronal cell death and synaptic loss, causes cognitive decline in patients with AD [36,37]. Moreover, several studies have demonstrated that PGE has neuroprotective effects against a variety of cytotoxic conditions [18,19,20,21]. Thus, to examine whether PGE affected neurodegeneration, we conducted immunofluorescence staining in the hippocampal formation in WT and 5XFAD mice using NeuN and SYN antibodies to evaluate neuronal cell death and synaptic degeneration, respectively (Figure 4A). Quantitative results revealed that optical density of CA1, CA3, and ML in the hippocampus strikingly decreased in 5XFAD mice treated with vehicle compared with WT mice treated with vehicle. PGE-treated 5XFAD mice showed a significant increase in optical density compared to that of vehicle-treated 5XFAD mice (Figure 4B–D). In addition, quantitative results showed that NeuN-positive cells in the subiculum were reduced in vehicle-treated 5XFAD mice compared to those in vehicle-treated WT mice. Moreover, PGE-treated 5XFAD mice exhibited a significant increase in NeuN-positive cells than vehicle-treated 5XFAD mice (Figure 4E). Therefore, these results show that PGE has an alleviative effect on neurodegeneration in the brains of 5XFAD mice.

### 3.5. Effect of PGE on Oxidative Stress in the Animal Models of AD 

**Figure 5:**
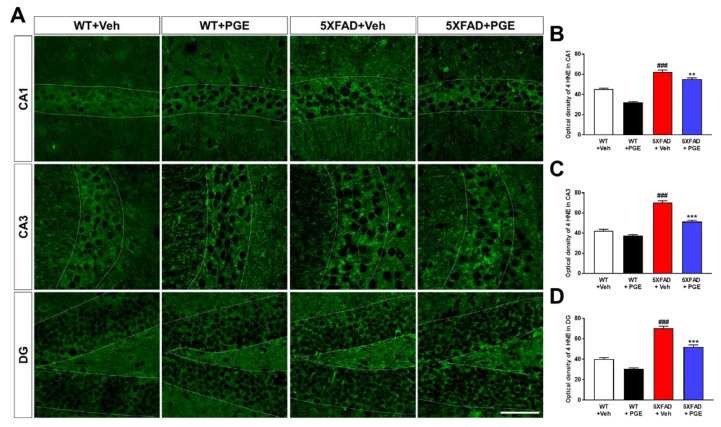
Immunofluorescence staining to exhibit the antioxidant effect of PGE on oxidative stress in the hippocampus of 5XFAD mouse model of AD. (**A**) Representative images display 4 hydroxynonenal (4 HNE) immunoreactivity in the hippocampus in WT and 5XFAD mice treated with vehicle and PGE, respectively. Quantitative analyses of oxidative stress are shown by an optical density of 4 HNE in (**B**) CA1, (**C**) CA3, and (**D**) DG of the hippocampus. The vehicle groups and PGE groups were all treated with the same volume of dissolved in saline or PGE, respectively. Data are presented as the mean ± S.E.M. Scale bar = 50 μm. ^###^ *p* < 0.001: Vehicle-treated WT mice (white bar) vs. vehicle-treated 5XFAD mice (red bar). ** *p* < 0.01 and *** *p* < 0.001: Vehicle-treated 5XFAD mice (red bar) vs. PGE-treated 5XFAD mice (blue bar). Statistical analysis between the four groups was performed by one-way ANOVA, followed by Turkey’s post hoc test (*n* = 30–32 images of brain section obtained from 4 mice in the WT + vehicle group, 5 mice in the WT + PGE group, 5 mice in the 5XFAD + vehicle group, and 5 mice in the 5XFAD + PGE group were used for the analysis).

Numerous studies have shown that Aβ plays a crucial role in inducing oxidative stress [1,38,39,40]. Oxidative stress, such as increased intracellular levels of ROS, induces neuronal cell death through damaging proteins, lipids, and DNA [41]. To investigate the antioxidant effect of PGE treatment, we performed immunohistochemical staining with antibodies against anti-4 hydroxynonenal (4 HNE), which is a marker for oxidative stress, in the hippocampus of 5XFAD mice (Figure 5A). Quantitative results indicated that the optical density of 4 HNE in the CA1, CA3, and DG were significantly increased in vehicle-treated 5XFAD mice than in vehicle-treated WT mice (Figure 5B–D). Interestingly, PGE-treated 5XFAD mice showed a remarkable decrease in the optical density of 4 HNE compared with vehicle-treated 5XFAD mice. These results demonstrate that PGE has an antioxidant effect in the brain with AD.

### 3.6. Effect of PGE on Aβ-Induced Cell Death and Oxidative Stress in Cultured Hippocampal Neurons

**Figure 6:**
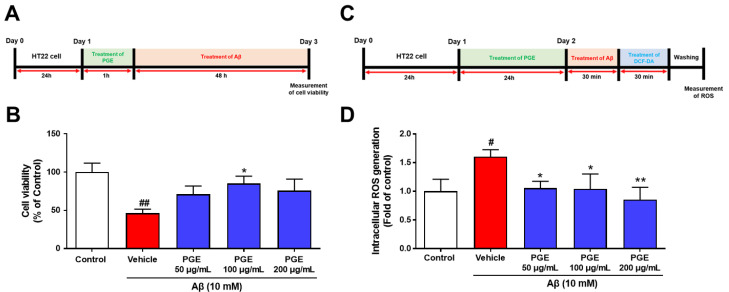
Inhibitory effect of PGE on Aβ_25–35_-induced oxidative stress in mouse hippocampal neuron cell line (HT22). (**A**) HT22 cells were treated to PGE (50, 100, and 200 μg/mL), and then incubated with 10 mM Aβ_25–35_ for 24 h. (**B**) The cell viability of the PGE (50 and 200 μg/mL) and Aβ_25–35_-treated HT22 cells was the trend to be higher than the vehicle and Aβ_25–35_-treated HT22 cells. The cell viability of the PGE (100 μg/mL) and Aβ_25–35-_treated HT22 cells was significantly higher than that of the vehicle and Aβ_25–35_-treated HT22 cells. (**C**) HT22 cells were treated with PGE (50, 100, and 200 μg/mL), and then incubated with 10 mM Aβ_25–35_ for 30 min. (**D**) Intracellular ROS of PGE (50, 100, and 200 μg/mL) and Aβ_25–35_-treated HT22 cells was significantly lower than that of vehicle and Aβ_25–35_-treated HT22 cells. The control, vehicle, and PGE groups were all treated with the same volume of saline or PGE, respectively. Data are presented as the mean ± S.E.M. ^#^ *p* < 0.05 and ^##^ *p* < 0.01: Control (white bar) vs. vehicle (red bar). * *p* < 0.05 and ** *p* < 0.01: Vehicle (red bar) vs. PGE (blue bar). Statistical analysis between the four groups was performed by one-way ANOVA, followed by Turkey’s post hoc test. All experiments were carried out in triplicate.

We observed the neuroprotective and antioxidant effects of PGE in the animal model of AD (Figure 4 and Figure 5). Thus, we conducted an in vitro experiment to confirm the direct neuroprotective and antioxidant effects of PGE against Aβ-induced neurodegeneration and oxidative stress. To confirm the neuroprotective and antioxidant effects of PGE, we used 3-(4,5-dimethylthiazol-2-yl)-5-(3-carboxymethoxyphenyl)-2-(4-sulfophenyl)-2H-tetrazolium (MTS) assay to assess cell viability. The HT22 cells were treated with Aβ (10 mM) and/or PGE (50, 100, and 200 µg/mL) (Figure 6A). The Aβ-treated HT22 cells showed about 50% reduction of cell viability. The PGE-treated hippocampal cells all showed some increase in cell viability, and particularly, a PGE concentration of 100 µg/mL significantly inhibited the Aβ-mediated reduction in cell viability (Figure 6B). Next, to investigate whether PGE can alleviate the Aβ-induced intracellular ROS, we performed the DCFH-DA assay (Figure 6C). Subsequently, HT22 cells were treated with Aβ (10 µM) and/or PGE (50, 100, and 200 µg/mL). Intracellular ROS generation increased about 1.7 times in the Aβ-treated group compared to that of the control but was significantly reduced when additionally treated with PGE (Figure 6D). These results show that PGE has a neuroprotective and antioxidant effect in Aβ-treated hippocampal neurons.

### 3.7. Effect of PGE on Neuroinflammation in the Brain of AD Transgenic Mice

**Figure 7:**
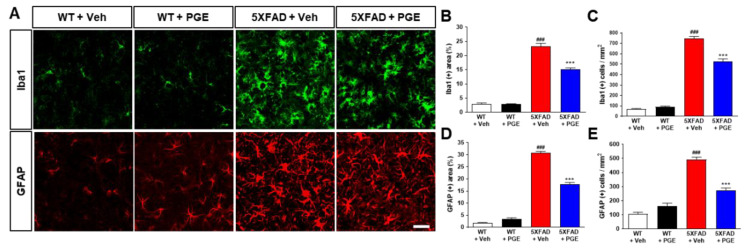
Immunofluorescence staining to show the inhibitory effect of PGE on neuroinflammation in the subiculum of 5XFAD AD mouse model. (**A**) Representative images indicate ionized calcium binding adapter molecule 1 (Iba1) and glial fibrillary acidic protein (GFAP) immunoreactivity in the subiculum of WT and 5XFAD mice treated with vehicle and PGE, respectively. Quantitative analyses of neuroinflammation are exhibited by (**B**) Iba1-positive area, (**C**) Iba1-positive cells, (**D**) GFAP-positive area, and (**E**) GFAP-positive cells. The vehicle groups and PGE groups were all treated with the same volume of saline or PGE, respectively. Values are expressed as the mean ± S.E.M. Scale bar = 50 μm. ^###^ *p* < 0.001: Vehicle-treated WT mice (white bar) vs. vehicle-treated 5XFAD mice (red bar). *** *p* < 0.001: Vehicle-treated 5XFAD mice (red bar) vs. PGE-treated 5XFAD mice (blue bar). Statistical analysis between the four groups was performed by one-way ANOVA, followed by Turkey’s post hoc test (30–32 images of brain section obtained from 4 mice in the WT + vehicle group, 5 mice in the WT + PGE group, 5 mice in the 5XFAD + vehicle group, and 5 mice in the 5XFAD + PGE group were used for the analysis).

Neuroinflammation is one of the main factors that affect neuronal cell death and cognitive dysfunction in the brain with AD [42,43]. PGE has been demonstrated to have anti-inflammatory effects [18,19,20,21]. Therefore, to ascertain whether PGE treatment affects neuroinflammation, we conducted immunofluorescence staining in the subiculum of WT and 5XFAD mice using GFAP or Iba1 antibodies, a marker of astrocytes and microglia, respectively (Figure 7A). Quantitative results indicated that the Iba1-positive area and cells in the subiculum were remarkably increased in vehicle-treated 5XFAD mice than in vehicle-treated WT mice. Moreover, PGE-treated 5XFAD mice displayed a reduction in the Iba1-positive area and cells compared with vehicle-treated 5XFAD mice (Figure 7B,C). Similarly, quantitative results showed that the GFAP-positive area and cells in the subiculum were markedly higher in 5XFAD mice treated with vehicle than in WT mice treated with vehicle. However, PGE-treated 5XFAD mice exhibited a decrease in GFAP-positive area and cells compared with vehicle-treated 5XFAD mice (Figure 7D,E). Collectively, our results suggest that PGE has an anti-inflammatory effect on the brains of 5XFAD mice.

### 3.8. Effect of PGE on Adult Hippocampal Neurogenesis in the Hippocampus of 5XFAD Mice

**Figure 8:**
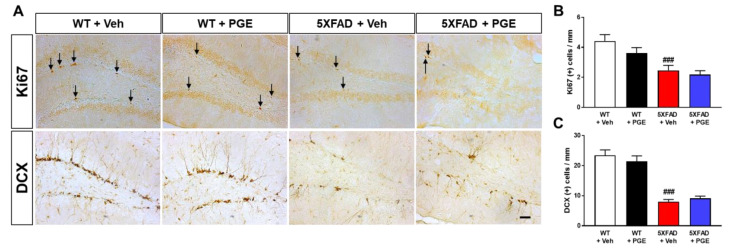
Immunohistochemical staining to indicate the effect of PGE on adult hippocampal neurogenesis (AHN) in the subgranular zone (SGZ) of DG in the hippocampus in 5XFAD AD mouse model. (**A**) Representative images exhibit Ki67 (Ki67 positive cells indicated with black arrows) and doublecortin (DCX) immunoreactivity in the SGZ of DG in the hippocampus of WT and 5XFAD mice treated with vehicle and PGE, respectively. Quantitative analyses of adult hippocampal neurogenesis are shown by (**B**) Ki67-positive cells and (**C**) DCX-positive cells. The vehicle groups and PGE groups were all treated with the same volume of saline or PGE, respectively. Values are expressed as the mean ± S.E.M. Scale bar = 50 μm. ^###^ *p* < 0.001: Vehicle-treated WT mice (white bar) vs. vehicle-treated 5XFAD mice (red bar). Statistical analysis between the four groups was performed by one-way ANOVA, followed by Turkey’s post hoc test (30–32 images of brain section obtained from 4 mice in the WT + vehicle group, 5 mice in the WT + PGE group, 5 mice in the 5XFAD + vehicle group, and 5 mice in the 5XFAD + PGE group were used for the analysis).

Cognitive function is closely associated with AHN [44]. In addition, several studies have shown that AHN levels are reduced in the AD brain, consequently impairing cognitive function [45,46]. To determine whether administration of PGE affects AHN, we performed immunohistochemical staining of the SGZ of DG in the hippocampus in WT and 5XFAD mice using Ki67 and DCX antibodies, markers of proliferating cells and neuronal precursor cells, respectively (Figure 8A). Quantitative results revealed that Ki67- and DCX-positive cells in the SGZ of DG in the hippocampus were significantly decreased in vehicle-treated 5XFAD mice than in vehicle-treated WT mice. However, there was no significant difference in AHN in 5XFAD mice treated with PGE compared with 5XFAD mice treated with vehicle for both Ki67 and DCX (Figure 8B,C). Consequently, our result found that PGE has not affected the enhancement of AHN in the AD animal model.

## 4. Discussion

**Figure 9:**
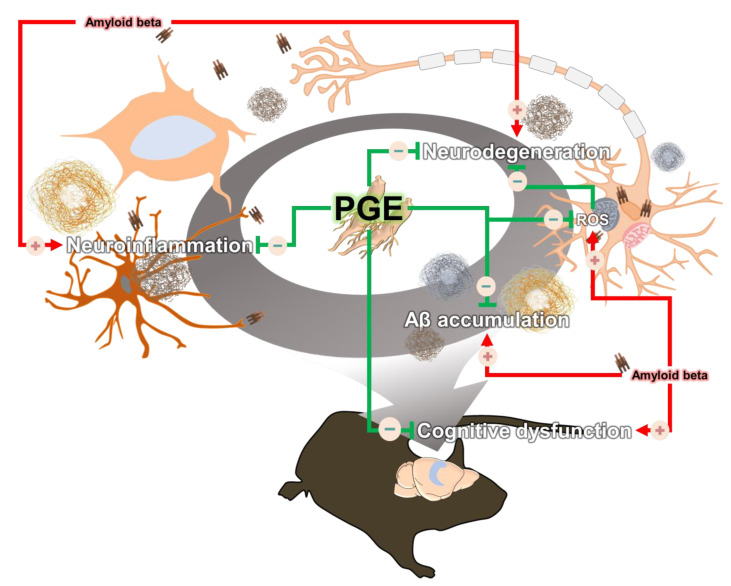
Schematic diagram of the ameliorative effect of PGE against Aβ-induced pathology and cognitive dysfunction. Black arrows indicate Aβ-induced pathologies and blue arrows display the amelioration effect of PGE administration in the brain of a 5XFAD mouse model. Up-regulation and down-regulation are indicated by a plus (+) and minus (−), respectively.

Since the pathogenesis of AD is induced by various pathogenic factors, there is increasing interest in multifunctional agents that could be used to treat AD such as natural products that act on multiple targets and have several bioactive effects [47,48]. Although PGE exhibits many beneficial biological effects in various diseases and symptoms, there are no studies on the therapeutic effects of PGE on cognitive dysfunction and AD pathology. In this study, we demonstrated the ameliorative effect of PGE on Aβ-related cognitive impairment in an AD mouse model (Figure 2). In addition, through histological analyses, we demonstrated the ameliorative effects of PGE against Aβ-mediated pathologies that cause AD-related cognitive decline (Figure 3, Figure 4, Figure 5 and Figure 7). Moreover, our in vitro result directly confirmed that PGE reduces Aβ-induced neuronal cell death and Aβ-induced oxidative stress (Figure 6). Furthermore, we found that the alleviative effect of PGE on memory impairments does not mediate the regulation of AHN in the brain of the 5XFAD mouse model (Figure 8). Taken together, our findings suggest that PGE, which acts by inhibiting Aβ-related pathology and ameliorating Aβ-induced cognitive impairment, could be a potential therapeutic agent for AD (Figure 9).

In this study, we found that PGE did not influence the regulation of AHN in the brain of 5XFAD mice (Figure 8). Consistent with our previous study [30], we confirmed that PGE did not affect AHN. Our studies had shown that PGE was not related to AHN in B6SJL/F1 mice at the age of 6 months (Figure 8) and C57BL/6 mice at 2 months of age [30]. Surprisingly, it has been reported that administration of PGE for 1 month increases the number of DCX-positive cells in 12-month-old healthy mice [49]. Although conflicting results exist regarding the effects of PGE on AHN [30,49], it is speculated that different treatment doses, total number, duration of administration, age of animals, and extraction methods displayed different effects. Since adult neurogenesis depends on age and AD progression [50], it is necessary to validate the effect of PGE on adult neurogenesis according to age and AD progression. However, these studies are not within the scope of the present study. Nevertheless, the reason why a positive effect on AHN was not observed in AD mice is that the hippocampus, which is important for learning and memory, is damaged at the early stages of AD [51]. Unfortunately, the 5XFAD model used in this study is a model that shows a rapid progression to AD [26]; hence, it corresponds to a later stage of AD that presents with a significantly massive accumulation of Aβ. Since the stage of AHN is observed after the hippocampus has already been significantly damaged, it is difficult to expect a dramatic positive effect of PGE on AHN.

The first possible mechanism by which to ameliorate AD pathogenesis might be associated with hormesis. Hormesis was defined as “the phenomenon of achieving the health-beneficial effects by exposure to mild stress” [52]. Mild exogenous and endogenous stressors are as follows: Irradiation, pro-oxidants, alcohols, and various bioactive components of plants [53]. Several studies have reported that hormesis, triggered by various bioactive components in plants, can show neuroprotective and antioxidant effect, and can relieve or prevent neuropathology in various neurodegenerative disorders [54,55,56,57]. Thus, it seems likely that the physiological active components of PGE may induce oxidative stress such as mild levels of ROS, reactive nitrogen species (RNS), and other free radicals. These phenomena induced by mild stressors consequently can induce disease-modifying effects by stimulating various adaptive mechanisms and pathways. The second possible mechanism might act via breaking the vicious circle induced by oxidative stress in the AD brain. It is well known that the brain, with its high oxygen consumption and lipid-rich content, is especially vulnerable to oxidative stress [58,59]. Surprisingly, there is compelling evidence demonstrating that oxidative stress is a prominent feature of AD linking the progression of neuronal dysfunction and death, exhibiting oxidative stress as a key pathogenic role in the exacerbation of AD [59,60,61,62,63]. In particular, over-production and accumulation of Aβ peptides play a crucial role in inducing oxidative stress and neuronal cell death [64,65]. Several studies reported that defects in antioxidant defense mechanisms can lead to increased oxidative stress and further accelerate the deposition of Aβ in AD transgenic mice [61,66,67]. The astrocytes and microglia activated by Aβ oligomers induce oxidative stress through the production of ROS and RNS [66], further provoking neuroinflammation [67,68]. The induced oxidative stress could disrupt neurite terminals and cell bodies, leading to synaptic loss and neuronal cell death [69,70]. Moreover, oxidative stress and inflammation response even interact with each other to exacerbate neurodegeneration [60,71]. Ultimately, oxidative stress might aggravate the production and aggregation of Aβ to promote neuroinflammation and neurodegeneration, leading to a vicious cycle of pathogenesis in AD. Numerous studies have previously demonstrated the antioxidative properties of PGE on various cell lines [17,72,73]. This compelling data allows us to deduce that the anti-amyloid, anti-inflammatory, and neuroprotective activities of PGE may eradicate the vicious circle induced by oxidative stress. The third possible mechanism might be related to modulating the imbalance between excitatory and inhibitory (E/I) neurotransmitters in the brain with AD. The balance of E/I neurotransmitter is a crucial factor in learning and memory, and imbalance between E/I neurotransmitter in an AD animal model underlies cognitive impairment at both the level of neural circuits and networks [74]. The imbalance of E/I neurotransmitter occurs in AD due to not only the dysfunction of the excitatory cholinergic and glutamatergic transmission but also the dysfunction of the gamma-aminobutyric acid (GABA) transmission [75,76]. Particularly, one of the studies reported that the amyloidogenic or non-amyloidogenic pathway is under the control of neuronal transmissions, such as acetylcholine, serotonin, norepinephrine, and dopamine, and the dysfunction of neuronal transmission accelerates the accumulation of Aβ by reducing control over the non-amyloidogenic pathway [77]. Interestingly, active compounds in PG are known to contain GABA, a neurotransmitter that is essential for brain energy metabolism [78]. The GABA in PG may improve cholinergic transmission in AD patients through activation of the GABA intracortical circuitry, a mechanism similar to homotaurine, a natural amino acid that mimics GABA found in seaweeds [79]. In addition, ethanol extract of PG has been reported to inhibit acetylcholinesterase (AChE) activity [25]. Considering the previous studies, PGE may exert the neuroprotective effect through the regulation of the imbalance between E/I neurotransmitters in the AD brain, such as activating the excitatory cholinergic and inhibitory GABAergic transmission.

Many components of PGE have a variety of activities in the brain [20,21,22,80,81]. It has been reported that platycodin D not only promotes neurite outgrowth of neuronal cells [30], but also protects neuronal cells against oxygen-glucose deprivation and ischemia [80,82]. In addition, platycoside E showed a cognitive-enhancing effect against ethanol-induced cognitive impairment in ethanol-treated mice [24]. Moreover, platycodin A exhibited a neuroprotective effect by inhibiting glutamate-induced toxicity [21] and anti-inflammatory effects by suppressing cytokine and chemokine secretion and NF-κB activation [83]. Furthermore, selenium polysaccharides rescue H_2_O_2_-mediated neuronal cell death by inhibiting oxidative stress [20]. Unfortunately, since we have not investigated the effects of each component of PGE, it is difficult to describe which component of PGE has this therapeutic effect. Nonetheless, PGE, which possesses a number of components with such beneficial effects, could be used as a therapeutic agent to improve AD-related pathology by acting as a multi-targeting agent for AD caused by various pathogenic factors. In this study, our results showed the antioxidant and neuroprotective effects of PGE against Aβ-induced neurotoxicity. In addition to PG, natural products with antioxidant properties such as olive oil, green tea, magnolia extract, grape, blueberries, and ginkgo biloba have also been reported to have antioxidant and neuroprotective effects in AD [84,85,86,87,88,89,90,91,92,93]. These natural products contain abundant natural polyphenols in common. Natural polyphenols have antioxidant properties that directly scavenge free radicals and activate antioxidant defenses [94,95]. In addition, natural polyphenols activate the Nrf2-antioxidant response element signaling pathway, which stimulates the synthesis of endogenous antioxidant molecules in cells [59,96]. Moreover, it also shows neuroprotective effects by modulating the activity of NF-κB or SIRT1 [97,98,99,100]. Since PGE contains 14 types of natural polyphenols [101], they could share a similar pathway to show antioxidant and neuroprotective effects.

## 5. Conclusions

Taken together, our data suggest that PGE treatment alleviates multiple aspects of neuropathology that correlate with cognitive improvement in AD, suggesting that PGE might be one of the therapeutic agents for AD. Therefore, we propose a study to investigate the effect of PGE on pathogenic factors other than Aβ in brains with AD-like pathology. Since we have investigated the effect of PGE on only Aβ-related pathology in female transgenic mice overexpressing Aβ, further studies are needed to elucidate the therapeutic efficacy of PGE in males and to reveal the effects of PGE on other factors, such as various type of cognitive function, hyperphosphorylated tau proteins, impaired long-term potentiation, alteration of the neuronal transmission, and mitochondrial dysfunction. Additionally, the use of biochemical analysis, such as Western blot and quantitative real time polymerase chain reaction, would be useful for understanding the molecular mechanism, as well as signaling pathway related to the beneficial effect of PGE.

## Data Availability

None.

## Supplementary Materials

The following are available online at https://www.mdpi.com/2076-3921/10/2/207/s1. Figure S1: Preparation of mouse brain tissues for immunoreactivity; Figure S2: The topographical analysis procedure for quantification of fluorescent signals in the mice brains; Table S1: Platycoside E and platycodin D contents of *Platycodon grandiflorum* extract; Table S2: Gene-specific primer sequences used for the present study.
