# Supplementary material for: Platycodon grandiflorum Root Protects against Aβ-Induced Cognitive Dysfunction and Pathology in Female Models of Alzheimer’s Disease
Source: Antioxidants (Basel). 2021 Feb 1;10(2):207. doi: 10.3390/antiox10020207 (PMC7912782; doi:10.3390/antiox10020207)
Supplement: Supplementary file 1 [file antioxidants-10-00207-s001.zip › Supplementary table.docx]

**Supplementary Table**

| Saponins | Retention time (min)* | Content (μg/g extract d.b.) |
| --- | --- | --- |
| Platycoside E | 14.246 | 942.32±97.97 |
| Platycodin D | 44.316 | 1597.14±21.71 |

**Supplementary Table S1**. Platycoside E and platycodin D contents of *Platycodon grandiflorum* extract. Retention times of standards (platycoside E and platycodin D) were 14.282 and 44.298 min, respectively.

| **Gene** | **Primer Sequence (5’→3’)** | **Gene Accession Number (Version)** | **Primer Name** | **Reference** |
| --- | --- | --- | --- | --- |
| APP | F: AGG ACT GAC CAC TCG ACC AG | NM_000484 (NM_000484.4) | oIMR3610 | [1] |
|  | R: CGG GGG TCT AGT TCT GCA T |  | oIMR3611 |  |
| PSEN1 | F: AAT AGA GAA CGG CAG GAG CA | NM_000021 (NM_000021.4) | oIMR1644 | [2] |
|  | R: GCC ATG AGG GCA CTA ATC AT |  | oIMR1645 |  |

**Supplementary Table S2**. Gene-specific primer sequences used for the present study.

**References**

1. Hendriks, L.; van Duijn, C.M.; Cras, P.; Cruts, M.; Van Hul, W.; van Harskamp, F.; Warren, A.; McInnis, M.G.; Antonarakis, S.E.; Martin, J.J., et al. Presenile dementia and cerebral haemorrhage linked to a mutation at codon 692 of the beta-amyloid precursor protein gene. *Nat Genet* **1992**, *1*, 218-221, doi:10.1038/ng0692-218.

2. Schellenberg, G.D.; Bird, T.D.; Wijsman, E.M.; Orr, H.T.; Anderson, L.; Nemens, E.; White, J.A.; Bonnycastle, L.; Weber, J.L.; Alonso, M.E., et al. Genetic linkage evidence for a familial Alzheimer's disease locus on chromosome 14. *Science* **1992**, *258*, 668-671, doi:10.1126/science.1411576.
