# Supplementary material for: Platycodon grandiflorum Root Protects against Aβ-Induced Cognitive Dysfunction and Pathology in Female Models of Alzheimer’s Disease
Source: Antioxidants (Basel). 2021 Feb 1;10(2):207. doi: 10.3390/antiox10020207 (PMC7912782; doi:10.3390/antiox10020207)
Supplement: Supplementary file 1 [file antioxidants-10-00207-s001.zip › Supplementary figure.docx]

**Supplementary Figures**


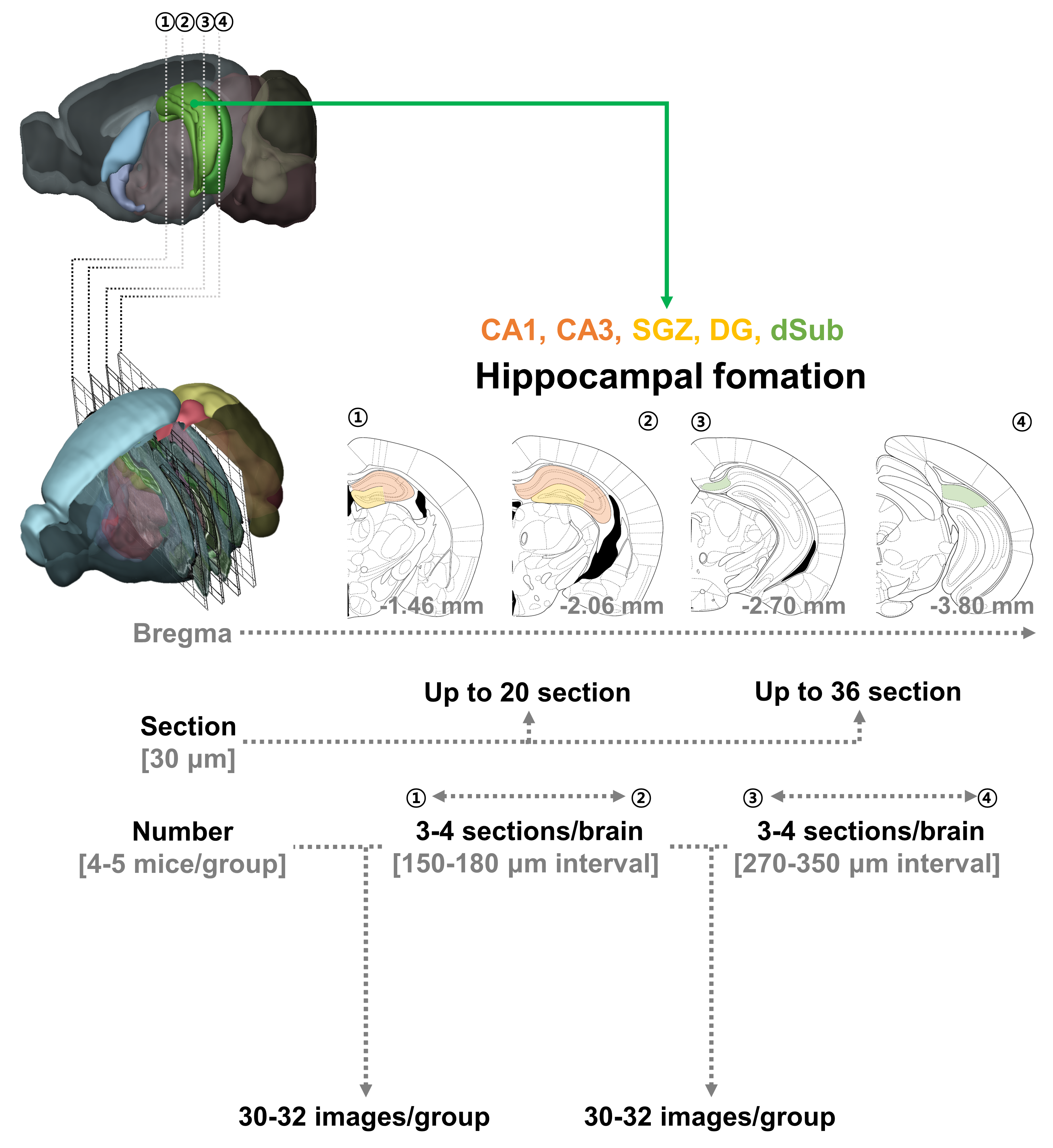


**Supplementary Figure S1.** Preparation of mouse brain tissues for immunoreactivity. From ① to ②, hippocampus such as CA1, CA3, and dentate gyrus (DG) was designated from -1.46 mm to -2.06 mm from the bregma. From ③ to ④, dorsal subiculum (dSub) was designated from -2.70 mm to -3.80 mm from the bregma. Fixed and cryoprotected mouse brains were coronally sectioned at a thickness of 30 μm in cryostat. twenty (hippocampus part) or thirty-six (dSub part) sections per mouse were obtained using the cryosection. In the hippocampus part, three to four sections per brain were taken from 4-5 mice at 150-180 μm intervals to obtain 30-32 images. In the dSub part, three to four sections per brain were taken from 4-5 mice at 270-350 μm intervals to obtain 30-32 images for dorsal subiculum. The acquired images were subjected to topographical quantification and statistical analysis in a blind manner. DG, dentate gyrus; dSub, dorsal subiculum; SGZ, subgranular zone.


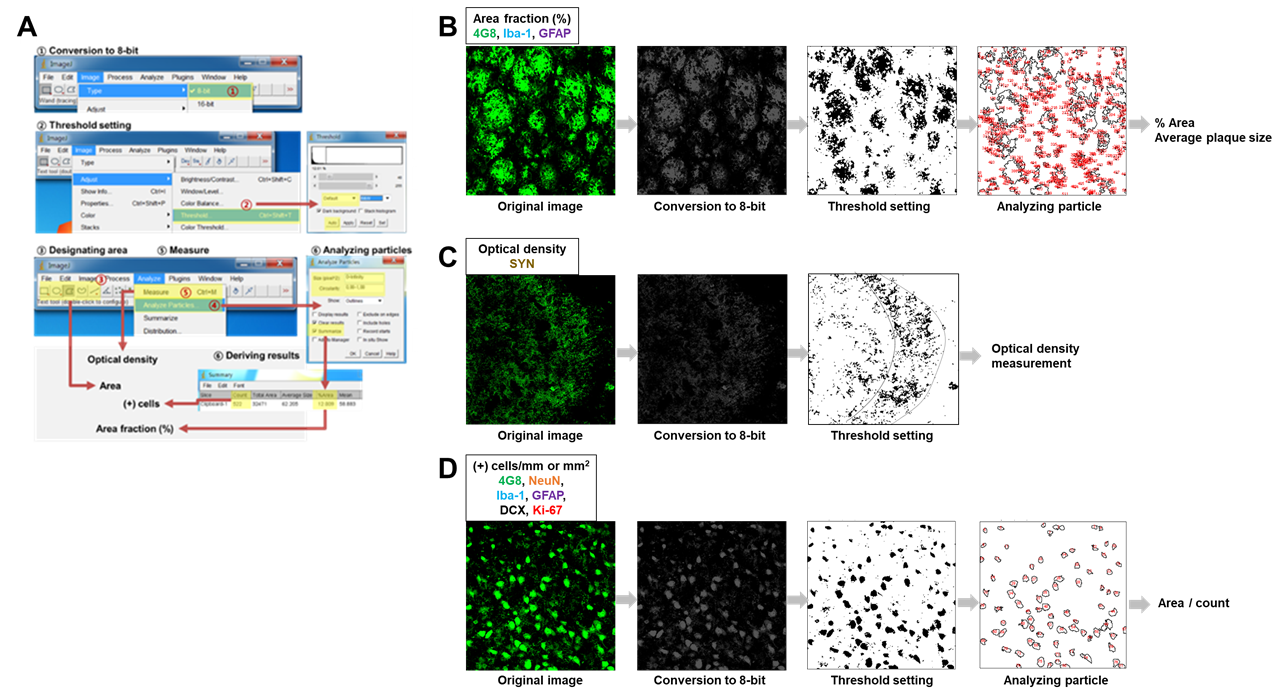


**Supplementary Figure S2.** The topographical analysis procedure for quantification of fluorescent signals in the mice brains. A) The procedures for the analysis steps for “area faction”, “optical density”, and “cells per area” in the ImageJ program. B) Quantitative procedure of “area fraction” applied for histological analysis of fluorescent signals of 4G8, Iba-1, and GFAP. Quantitative procedure of “average plaque size” applied for histological analysis of 4G8 C) Quantitative procedure of “optical density” applied for histological analysis of SYN-immunoreactivity. D) Quantitative procedure of “positive cells/area” applied for histological analysis of NeuN, Iba-1, GFAP, 4G8, DCX, and Ki-67-immunoreactivity.
